# Gene Editing with CRISPR/Cas Methodology and Thyroid Cancer: Where Are We?

**DOI:** 10.3390/cancers14030844

**Published:** 2022-02-08

**Authors:** Cesar Seigi Fuziwara, Diego Claro de Mello, Edna Teruko Kimura

**Affiliations:** Department of Cell and Developmental Biology, Institute of Biomedical Sciences, University of Sao Paulo, Sao Paulo 05508-000, Brazil; mellodc@usp.br

**Keywords:** thyroid cancer, gene editing, CRISPR/Cas, Cas9, CRISPRi, CRISPRa, gene modulation

## Abstract

**Simple Summary:**

The advent of genomic editing with CRISPR/Cas9 has transformed the way we manipulate the genome, and has facilitated the investigation of tumor cell biology in vitro and in vivo. Not only we can modify genome sequence to blunt an overactivated gene or correct a mutation, but also we may modulate gene expression using CRISPR/Cas system. In this review, we present the basics of CRISPR/Cas methodology, its components and how to start a CRISPR/Cas experiment; Moreover, we present how CRISPR/Cas methodology has been applied to study the function of coding and noncoding genes in thyroid cancer and provided insights into cancer biology.

**Abstract:**

Important advances on the role of genetic alterations in thyroid cancer have been achieved in the last two decades. One key reason is linked to the development of technical approaches that allowed for the mimicking of genetic alterations in vitro and in vivo and, more recently, the gene editing methodology. The CRISPR/Cas methodology has emerged as a tangible tool for editing virtually any DNA sequence in the genome. To induce a double-strand break and programmable gene editing, Cas9 endonuclease is guided by a single-guide RNA (sgRNA) that is complementary to the target sequence in DNA. The gene editing per se occurs as the cells repair the broken DNA and may erroneously change the original DNA sequence. In this review, we explore the principles of the CRISPR/Cas system to facilitate an understanding of the mainstream technique and its applications in gene editing. Furthermore, we explored new applications of CRISPR/Cas for gene modulation without changing the DNA sequence and provided a Dry Lab experience for those who are interested in starting “CRISPRing” any given gene. In the last section, we will discuss the progress in the knowledge of thyroid cancer biology fostered by the CRISPR/Cas gene editing tools.

## 1. Introduction

Gene editing using the CRISPR/Cas methodology has emerged as a promising tool to modify nearly any target DNA sequence in a genome, and has rapidly become an alternative to time-demanding methodologies such as zinc fingers nuclease (ZFN) and TALE nucleases (TALEN) [1]. In fact, the number of publications with “CRISPR” or “CRISPR/Cas” in the Pubmed database surpassed publications with “TALEN” or “Zinc-finger nucleases” by more than tenfold, in less than 10 years of the technique’s first description [2]. In this review, we first explore the fundamentals of the CRISPR/Cas system to facilitate an understanding of the technique and its application in gene editing. Moreover, we describe new applications for CRISPR/Cas in gene modulation without altering the DNA sequence and provide a Dry Lab Section 3 that allows readers to start “CRISPRing” their favorite genes. In the last section, we discuss how the CRISPR/Cas methodology was applied in the understanding of thyroid cancer biology.

## 2. The Principle of CRISPR/Cas

### 2.1. What Is CRISPR/Cas?

The CRISPR/Cas system is an endogenous RNA-guided prototype of immune defense against viral infection (DNA or RNA) in bacteria and archaea, initially described as a peculiar region of repetitive sequences detected downstream of the *Iap* gene’s translation termination codon in *E. coli* [3]. The CRISPR locus is composed of palindromic sequences of 14bp (repeats) that are interspaced by a 32-nucleotide (32-nt) sequence and repeated several times in the genome. The Cluster of Regularly Interspaced Palindromic Region (CRISPR), as it was named in 2002 [4], contains sequences of foreign DNA incorporated between the palindromic repeats (CRISPR array region). This system constitutes a prototype of adaptive immune responses by storing information of previous infections in the DNA, which is transcribed into CRISPR RNAs (crRNAs) [5].

As an essential part of the system, CRISPR-associated genes (Cas) are located adjacent to the CRISPR region and participate in several steps of adaptive immunity, in a process defined by the following three phases: adaptation, expression, and interference [6]. In the adaptation phase, specific Cas genes (i.e., Cas1 and Cas2) recognize the invading DNA, which is fragmented and incorporated into the CRISPR locus spacer region. In the expression phase, the CRISPR array is transcribed into crRNA, a small RNA that contains a 20-nt specific sequence (targeting the foreign DNA) and a segment of repeats that interacts with trans-activating crRNA (tracrRNA). This RNA structure is loaded into the Interference Cas protein, Cas9. In the interference phase, Cas9 endonuclease is complexed with crRNA + tracrRNA and targets foreign DNA based on a perfect complementarity match to the 20-nt of crRNA, which leads to the cleavage of the double-strand DNA due to the nuclease activity of Cas9.

**Figure 1:**
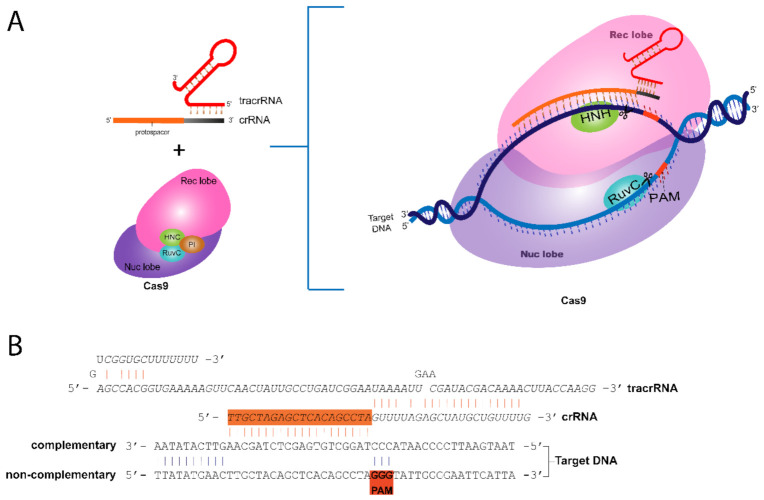
The basic components CRISPR/Cas9 system. (**A**) The crRNA associated with tracrRNA enters Cas9 endonuclease and guides the protein throughout the foreign DNA in search of a full-complementarity region adjacent to a PAM sequence in order to produce the double-strand break. (**B**) Detailed view of interaction of crRNA with the target DNA sequence (complementary sequence) and PAM sequence in the non-complementary strand of DNA.

The existence of an array of differences in the CRISPR/Cas loci structure in bacteria and archaea led to the classification of CRISPR systems based on the interference phase’s effector proteins. The current classification divided CRISPR into two classes and six types with particular Cas proteins [7]. The main difference between Class 1 and Class 2 is the composition of the Cas protein that participates in the interference Phase. In Class1, the CRISPR system recruits a large complex composed of several Cas proteins in the interference phase (Types I, III, and IV). On the other hand, in Class2 CRISPR, a single protein exerts multiple functions required for the interference phase (Types II, V, and VI). The focus of this review is the Cas9 protein, the most used Cas endonuclease for gene editing purposes. Additionally, Cas9 is a member of Class2 Type II CRISPR, in which a single protein is responsible for the interaction with guide RNA (crRNA + tracrRNA), DNA scanning, and double-strand break due to nuclease activity of the RuvC and HNH nuclease domains (Figure 1).

Understanding the principle of RNA-guided DNA cleavage by the CRISPR/Cas system in bacteria led to the repurposing of Cas9 to target DNA in eukaryotes, thus envisioning gene editing applications for functional investigation. In this new context, the single multifunctional protein Cas9 emerged as a catalyzer of gene editing with CRISPR/Cas due to the relative simplicity of expressing a single protein allied to the sgRNA (and other requirements, which we explore next).

### 2.2. How Is CRISPR/Cas Programmable?

One of the issues of gene editing prior to CRISPR/Cas was how to generate a programmable, targeted double-strand DNA break in the desired region. In 2012, a seminal Nobel Prize winning paper demonstrated the CRISPR/Cas system’s programmability [2]. This study showed CRISPR/Cas9 could be directed to any given DNA sequence upon the use of a single-guide RNA (sgRNA) formed by the fusion of crRNA + tracrRNA. The original bacteria/archaea CRISPR/Cas system relies on the expression of crRNA from the CRISPR array region that interacts with tracrRNA, a small 24-nt RNA that facilitates the maturation of crRNA by RNAIII nuclease [8] and interacts with Cas9. The mature form of crRNA (crRNA + tracrRNA) provides the 20-nt sequence that specifically targets foreign DNA sequence, while the tracrRNA recruits and activates Cas9 through conformational changes [2] (Figure 1).

**Figure 2:**
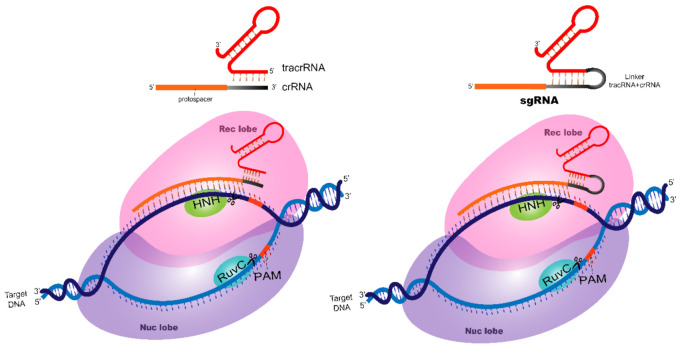
Structural differences among guide RNAs. Comparison of the crRNA associated with tracrRNA and the single-guide (sgRNA) structure. The junction of crRNA + tracrRNA with the linker loop resulted in the structure of single-guide RNA (sgRNA).

Artificial changes to link both crRNA + tracrRNA structures resulted in the chimeric sgRNA that simplified the structural elements necessary for sgRNA loading in Cas9 (Figure 2). This modification resulted in a simple system in which the 20-nt DNA sequence specific for the target gene is cloned into a plasmid that transcribes the single-guide RNA (explored in the Dry Lab Section 3).

However, when selecting the target 20-nt sequence to produce the sgRNA, an additional feature is essential, namely, the presence of protospacer-adjacent motif (PAM) sequence next to the target DNA in the genome. PAM is a short-conserved sequence motif (2–5 bp) essential for Cas9 activity that varies among species (Table 1). For example, the most commonly used Cas9, the SpCas9 from *Streptococcus pyogenes*, recognizes a PAM sequence of NGG one base pair downstream of the 20-nt target site in the non-complementary strand [2] (Figure 1). Indeed, the versatility of PAM sequences in Class 2 Type II CRISPR systems contributes to the programmability of CRISPR/Cas to virtually any sequence in the DNA.

### 2.3. Why Cas9?

There are different types of Cas protein in the Class 2 system, in which the same Cas protein, for example Cas9, exerts the function to bind to the sgRNA, unfold the double-strand, and cut the DNA. It is reasonable to infer that this type of Cas protein is better suited for gene editing purposes because the delivery of one single protein with a sgRNA is easier than delivering multiple proteins, as in the Class 1 Cas system.

Furthermore, Cas9, specifically SpCas9, is a 1368aa multi-domain protein composed of two lobes, the alpha-helical recognition (REC) lobe and the nuclease (NUC) lobe containing the HNH and the RuvC nuclease domains [14]. The following functions are performed by Cas9: (1) interacts with sgRNA; (2) scans DNA; (3) unfolds DNA double-strand, and (4) cuts both strands of DNA. 

Part of the popularity of SpCas9 is due to its PAM sequence NGG, which is simple and may be present in the majority of targets. However, SpCas9 is considered a large protein, with the coding sequence occupying most of the plasmid cloning space and impairing the cloning of multiple sgRNAs. In this context, researchers have investigated several types of bacteria and archaea genomes in search of new Cas proteins in Type II, V and VI groups, which may be smaller and even more specific (reduced off-targeting) than Cas9 (Table 1). Off-target is an unwanted DNA cleavage at unintended DNA sites due to the partial complementarity of sgRNA 20-nt sequence (usually with few mismatches) [15], that can lead to genomic instability and disable otherwise normal genes. Thus, the biochemical structural studies have focused on understanding the Cas9 protein structure to map and hack its structure to create new versions that are more specific as nucleases with reduced off-targeting. 

### 2.4. Mechanism behind Gene Editing

The Cas endonucleases (i.e., Cas9, Cas12, Casx, etc.) generate a DNA double-strand break at the target region guided by the sgRNA. As this type of DNA break is extremely deleterious because it may lead to loss of DNA content, cells express a DNA damage repair machinery to reunite DNA proficiently. The gene editing per se, meaning changes in the DNA sequence by insertion or deletion of nucleotides, occurs in this process of repairing (ligating) DNA broken strands. 

**Figure 3:**
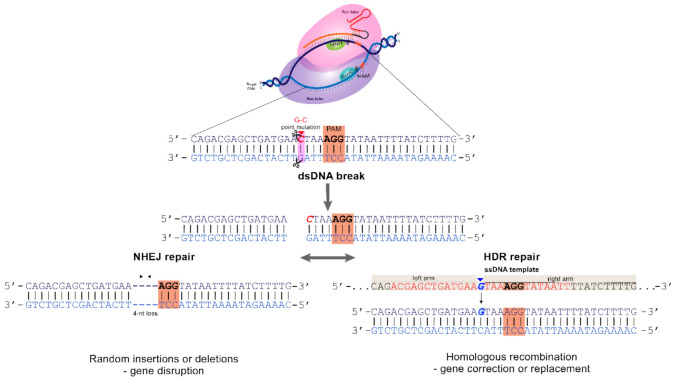
DNA repair mechanism. Mechanisms of gene editing mediated by NHEJ and HDR after CRISPR/Cas-induced double-strand break.

DNA double-strand breaks can be repaired through the following two main mechanisms: (1) Nonhomologous End Joining Repair (NHEJ) or (2) Homologous Directed Repair (HDR) (Figure 3).

NHEJ is the main mechanism for double-strand break repair because it is proficient at reuniting double-strand break ends. However, it may be unspecific and error-prone, and this property is especially important for gene disruption because it frequently causes microdeletions or insertions that ultimately lead to a loss of protein expression by frame shifting and premature stop-codon insertion.

On the other hand, HDR occurs less frequently (mainly in the cell cycle’s S-G2 phase) and uses endogenous (sequence in the sister chromosome) or exogenous homologous pieces of DNA to ligate double-strand breaks correctly without error. When using an exogenous sequence, a DNA template may contain the gene’s correct sequence to correct a mutation through HDR, for example, and must be provided with Cas9 + sgRNA. Nevertheless, a lower frequency of HDR (compared to NHEJ) is a scenario that may be improved by cell synchronization, drug treatment, or molecular biology manipulation to favor gene correction [16].

### 2.5. New Applications for CRISPR/Cas: Tailoring Cas Activity

Since the groundbreaking report of CRISPR/Cas9-mediated gene editing [2], intense investigations of Cas9 protein crystallography and biochemistry have resulted in important advances in the understanding of endonuclease function. It also permitted repurposing Cas9 variants into new applications such as CRISPRi or CRISPRa (explored later in this section) that perturb gene expression without changing the DNA sequence (epigenetic modulation) by inactivating or activating gene transcription. 

The nuclease activity of Cas9 generates a double-strand DNA break and can be modulated through specific mutations in the nuclease domain. The RuvC (D10A) or HNH (H840A) mutations individually render Cas9 enzyme single-strand break activity (called nickase: Cas9n), meaning it cuts only one strand of DNA. An endogenous repair system easily repairs this type of DNA damage; however, the Cas9n system can be used for gene editing in a double-sgRNA strategy (Table 2), where a double-strand break occurs only in the DNA region targeted by a pair of sgRNA complementary to a different strand of DNA, and requires an optimal distance (minus 4 to 40-nt). This strategy reduces off-targets because only on-targeting results in double-strand breaks [17], and is better exemplified in Section 4.2. Targeting Noncoding Genes in Thyroid Research.

**Figure 4:**
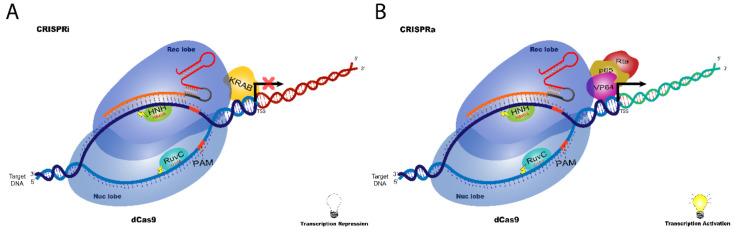
Gene modulation with CRISPR/dCas9 system. Dead Cas9 (dCas9) as a tool to modulate gene expression without gene editing. (**A**) In CRISPRi (interference) system, dCas9 is fused to transcriptional inhibitors such as KRAB that blocks transcription of sgRNA targeted region; (**B**) In the CRISPRa (activation) system, dCas9 is fused to transcriptional activators such as VP64, RTA and P65 to induce the expression of sgRNA targeted region.

The double-mutant (RuvC D10A and HNH H840A) is considered a “dead” Cas9 (dCas9) that does not cleave or nick the DNA strand, but it still interacts with target DNA region guided by the sgRNA. This property is especially noteworthy in disturbing gene expression when dCas9 impairs the recruitment of transcriptional complexes or blocks RNA polymerase [21]. In this context, dCas9 may be fused to different protein domains that can result either in gene expression silencing or gene induction [22], which we explore below in detail (Figure 4).

CRISPR interference (CRISPRi): the presence of dCas9 complex (dCas9 + sgRNA) in a coding region interferes with transcriptional elongation, RNA polymerase binding, or transcription factor binding leading to reduction of gene transcription [21]. In this study, the authors directed dCas9 + sgRNAs to the coding region of *mRFP* (mouse red fluorescent protein) and observed that the sgRNA targeting of the coding region’s proximal sequences (in the non-template strand) resulted in a 10 to 300-fold inhibition of gene expression. Moreover, targeting the proximal promoter (minus 35-nt box region) resulted in a strong knockdown of *mRFP* gene expression [21].

Further study of this mechanism led to the development of the CRISPRi methodology that relies on the use of dCas9 fused to the repressive chromatin-modifier domain in the N-terminal region, such as Krüppel-associated box (KRAB) of Kox1, to improve CRISPRi silencing efficiency [22]. Indeed, targeting the dCas9-KRAB complex (+sgRNA) to the proximal promoter or the first exons of *EGFP* (enhanced green fluorescent protein) resulted in robust inhibition of gene transcription (~15-fold) because KRAB recruited chromatin modifiers to proximal regulatory elements [22]. 

CRISPR activation (CRISPRa): In the CRISPRa system, dCas9 protein fused transcriptional activators, such as VP64 (tetrameric repeat of VP16 from Herpes virus) or the p65 activation domain, induces target gene expression [22] (Figure 4B). Indeed, targeting sgRNA to the upstream activation sequence of the *Gal4* gene, for example, resulted in a 25-fold and 12-fold activation for dCas9-VP64 and dCas9-p65, respectively. Further optimization of this system led to the discovery that the tandem fusion of VP64, p65, and Rta, in this order, generates a hybrid VP64-p65-Rta activator (called as VPR), which improves the activation efficiency in the CRISPRa system [20].

Both methodologies, CRISPRi and CRISPRa, have been validated to modulate the target gene transiently without modifying the DNA structure but the constant need for dCas9 + sgRNA expression in the cell to maintain gene modulation is a drawback. This fact can be overcome by using lentiviral delivery to express dCas9 and sgRNA stably, as mentioned in the seminal studies [20,21,22].

## 3. Dry-Lab: How Do I Use CRISPR In Vitro? 

To start a gene editing experiment with CRISPR/Cas9, two essential components are mandatory: (1) Cas9 (or other Cas endonuclease) and (2) sgRNA. A third optional component is a selection marker that facilitates the screening. The delivery of CRISPR/Cas9 (containing sgRNA) frequently uses a plasmid system that can be easily transfected into cells, which is explored below.

It is necessary to express Cas endonuclease (Cas9, for example) and sgRNA simultaneously to achieve DNA double-strand break, single-strand break or gene modulation (in this case, dCas9 + sgRNAs expression should be kept constant). One of the easiest methods is to clone the sgRNA sequence under the control of the U6 promoter in a plasmid that also expresses Cas9 endonuclease (or other Cas variants, discussed in the upper sections) and a selection marker, such as puromycin resistance, or a fluorescent reporter such as GFP. The Zhang Lab [18] constructed these plasmids, which are available in Addgene as PX459 or PX458, respectively, for Cas9 + puromycin resistance, or Cas9 + GFP. We use these plasmids as examples.

**Figure 5:**
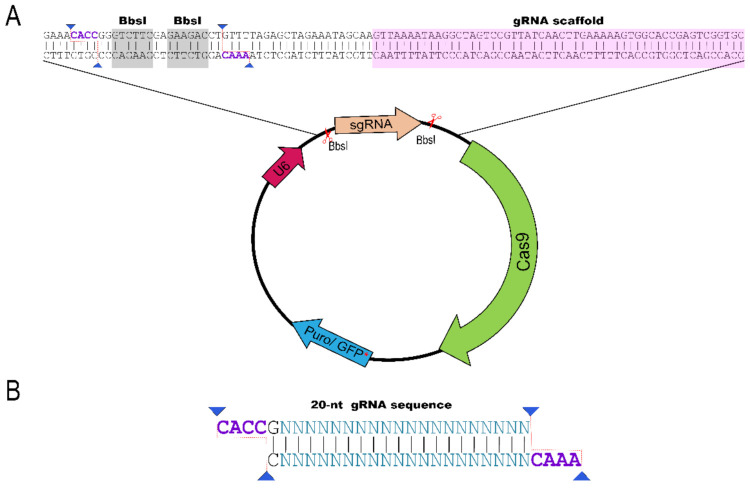
Plasmidial system to express CRISPR/Cas9 system. (**A**) CRISPR/Cas9 plasmid contains a site for BbsI digestion that creates the cloning site for the 20-nt sequence sgRNA; (**B**) The sgRNA sequence is ligated into BbsI-digested plasmids PX458 (GFP) or PX459 (puromycin resistance) as annealed DNA oligonucleotides with cohesive ends as shown in the figure CACC in the 5′ and CAAA in the 3′.

The cloning methodology is user-friendly and uses the restriction site created by the digestion of the plasmid PX459 or PX458 with BbsI endonuclease for sgRNA cloning as shown in Figure 5. The resulting open linear plasmid has cohesive ends with a 5′-CACC overhang and a 3′-CAAA overhang for ligation with sgRNA (DNA sequence). We recommend testing at least three different sgRNAs sequences in order to select the most efficient.

The sgRNA sequence can be efficiently designed using available online tools, such as ChopChop [23] or Gene Perturbation Platform (GPP)/CRISPick [24] that have algorithms to predict the efficiency of double-strand break and score sgRNAs according to the off-target prediction. These tools are especially efficient for coding-genes. After choosing the sgRNAs, the sequence is ordered as a pair of DNA oligonucleotides that contain the 20-nt target region (PAM sequence should be removed from the sgRNA sequence) and the cohesive ends to ligate into the BbsI digested plasmid (PX458 or PX459, for example) when these oligonucleotides are annealed to form a double-strand DNA (Figure 5).

**Figure 6:**
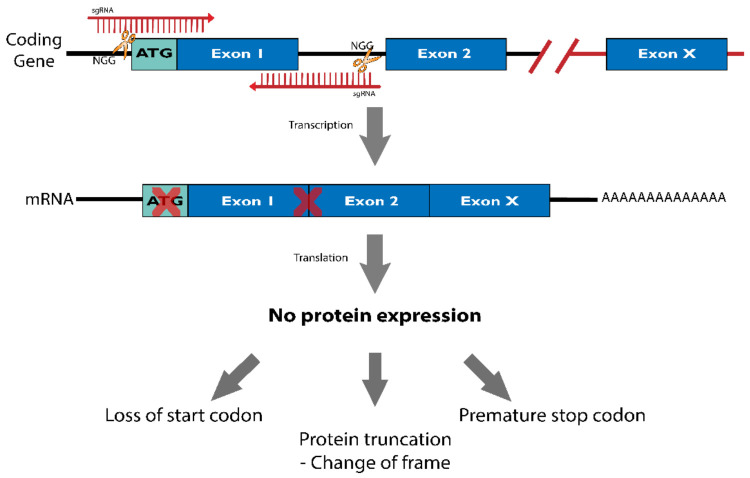
Editing protein-coding genes with CRISPR/Cas9. Targeting protein coding genes with CRISPR/Cas9-mediate gene editing. The objective is to disrupt the coding sequence using sgRNAs targeting the first exons of the gene, close to the start codon “ATG”. As a result, loss of protein expression is expected due to deletions or insertions that may disrupt the start codon, change protein frame or even insert a premature stop codon.

In the coding-gene context (Figure 6), the sgRNA design should preferentially favor the targeting of the coding-gene’s first exons, closer to the start codon region (ATG), if possible. This strategy aims to disrupt the coding sequence from the start codon and, therefore, truncate the protein or create a premature stop codon.

**Figure 7:**
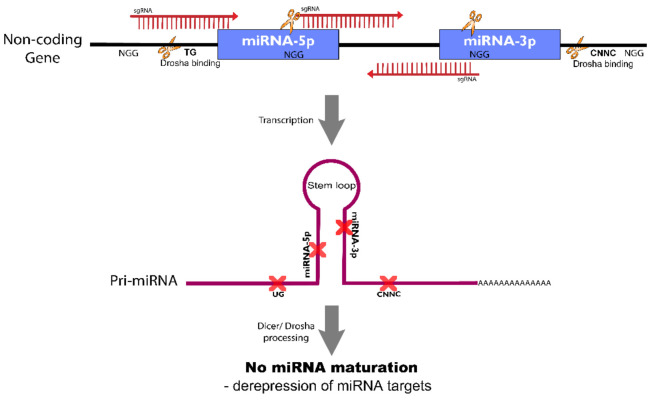
Editing non-coding genes with CRISPR/Cas9. Targeting non-coding genes with CRISPR/Cas9. In the figure the non-coding miRNA gene is represented and the sgRNAs target structural regions of the primary miRNA structure that are necessary for miRNA processing by DROSHA and DICER endonucleases.

In terms of targeting noncoding RNAs, the main objective is to disrupt structural or regulatory regions. For example, when targeting a microRNA(miRNA) gene (explored in Section 4.2 targeting noncoding genes in thyroid research), the sgRNA may be directed to the desired strand of miRNA (either 5p or 3p) to remove its DNA sequence. Alternatively, sgRNAs may target the structural regions of primary or precursor miRNA processing, for example DROSHA/DICER binding sites or stem loop region (Figure 7).

After plasmid sequence confirmation, cells are transfected and selected for the marker (GFP or puromycin) to generate a mixed population. This mixed population can be initially screened for the target gene or protein expression (dependent upon if it is a coding or noncoding gene). Moreover, the genomic DNA can be used to assess the extent of gene editing by a surveyor assay or T7 endonuclease assay (both assays use nucleases that recognize DNA annealing mismatch).

Next, if cells present gene editing (DNA or protein levels), clonal expansion by limiting dilution may be used to generate single cell clones for cleaner results. It is important to note that several clones should be derived from a single population to avoid genetic drifting. Again, gene and protein expression may be used to validate the efficacy of gene editing, but the DNA sequencing of the target region is mandatory to show the extent of gene editing.

Up to now, we described the most used strategy to deliver CRISPR/Cas9 + sgRNA to the cells, which is the plasmid system. Nevertheless, researchers are moving to the transient presence of Cas9 + sgRNAs in the cells to minimize the off-targeting effect, and this purpose may be achieved using ribonucleoprotein (RNP) transfection. RNPs consist of the protein Cas9, which can easily be purchased from several companies (either Cas9, Cas9n or dCas9 are available), complexed in vitro with the sgRNA. In this case, sgRNA can also be purchased from companies but may be transcribed in vitro. Then, Cas9 + sgRNA RNPs are delivered to the cells by conventional transfection or electroporation. The main advantage is that the Cas9 protein complex (+sgRNA) is readily available to the cells. On the other hand, in the plasmid system, Cas9 and sgRNA are first transcribed, and Cas9 is translated in the cytoplasm and transported back to the nucleus, where it can interact with sgRNA and target DNA.

## 4. Gene Editing with CRISPR/Cas in Thyroid Cancer Research

The thyroid cancer field has further developed by using gene editing with CRISPR/Cas due to the relative straight-forwardness of the methodology compared to other gene-editing platforms such as Zinc-fingers nucleases or TALEN [1]. One of the cornerstones to investigate a gene function is to blunt its expression. Unlike RNA interference, which requires the expression of short-interfering segment to maintain gene knockdown, the gene editing alters the DNA and leads to a stable modification of genome. Consequently, when this segment is transcribed it contains changes in RNA sequence that may modify the coding sequence of a mRNA, or the structure of a noncoding RNA. 

In this section, we discuss studies that used CRISPR/Cas to target coding and non-coding genes for the investigation of thyroid cancer biology. It is important to note that although we tried to organize the studies according to the target gene function or signaling pathway, most of the results cross-talk with each other. 

### 4.1. Targeting Coding Genes in Thyroid Cancer

#### 4.1.1. MAPK Pathway

Thyroid cancer is the most frequent endocrine cancer [25], and arises from two distinct main parenchymal cells, the thyroid-hormone producing follicular cells and the calcitonin-secreting C-cells (also called parafollicular cells). The majority of thyroid cancer is derived from follicular cells, and classified as well-differentiated (papillary and follicular) and undifferentiated (anaplastic) types, which are the focus on the present review, whereas the C-cells derived cancer, the medullar thyroid cancer, is less prevalent [26].

The most frequent histotype of thyroid cancer is the papillary thyroid cancer (PTC, approximately 80–85%) which in general exhibits a low mortality. On the other hand, the less common histotype, the anaplastic thyroid cancer (ATC, 1%) presents an extremely aggressive clinical behavior. The mitogen-activated protein kinase (MAPK) pathway contains the majority of driver oncogenes in both of the subtypes [27], and additional alterations, such as in *TERT* promoter and *TP53* genes in ATC [28,29]. In PTC, the MAPK oncogenes are the highly mutated *BRAF* (60–70%) gene, and less frequently the mutations in the isoforms of *RAS*, and the *RET* gene translocations (*RET/PTC* rearrangement), alterations that lead to overactivation of this signaling [30,31,32]. Besides the proliferative effect, MAPK signaling is involved in the control of cell differentiation, motility, invasion, apoptosis, and noncoding signaling [33,34,35,36].

BRAF^V600E^ point mutations are the most common event in PTC (~60%) and are detected in poorly differentiated thyroid cancer (PDTC) and ATC, approximately 33% and 45%, respectively [32,37]. Classically, mouse models BRAF^V600E^ targeted to thyroid cells develop PTC with high penetrance and facilitated important discoveries in thyroid-cancer development and progression [38,39,40]. However, even in the CRISPR era, mouse genetic manipulating is still a laborious and time-consuming process. In this context, zebrafish constitute an alternative model that can be easily manipulated in vitro and can facilitate rapid evaluation of the effects of genetic manipulation, as the development of major organs occurs within 5 days. Indeed, a zebrafish BRAF^V600E^ model (named Tg-BRAF-TOM) showed that BRAF activation in thyroid cells induced severe thyroid gland disorganization and impairment of thyroid hormone production as soon as 5 days postfertilization [41]. Moreover, adult Tg-BRAF-TOM animals displayed thyroid cancer that resembled human histopathological features. The RNAseq analysis revealed the activation of the epithelial-mesenchymal transition (EMT) program (as observed in larvae), and gene-set-enrichment analysis identified the transcription factor *twist3* (orthologue of *TWIST2*) when Tg-BRAF-TOM profile was compared to human PTC in TCGA database [32]. Targeting the *twist3* gene with CRISPR/Cas9-mediated gene editing resulted in a loss of function of the twist3 in zebrafish larvae and led to the partial restoration of thyroid follicles in Tg-BRAF-TOM animals revealing the potentiality of the technique in vivo [41].

Despite the classical MAPK genetic alteration, the gene expression of key MAPK genes were also detected in thyroid cancer. For example, the overexpression of epidermal growth factor receptor (EGFR) is reported in the thyroid cancer database from The Cancer Genome Atlas (TCGA) [32] and in aggressive ATC [42]. Indeed, CRISPR/Cas9-mediated *EGFR* gene editing in ATC cell line *SW579* resulted in EGFR knockout at the protein level that blocked ERK and AKT phosphorylation [42]. Moreover, EGFR loss induced cell growth arrest in the S-phase, and reduced colony formation ability. 

In the context of negative regulators of MAPK signaling, the NF2 protein (also known as Merlin) is a scaffold protein that regulates the receptor-mediated signaling pathway, such as MAPK and Hippo signaling [43]. The loss of expression or mutations in *NF2* have been detected in thyroid cancer and are linked to Ras-induced tumorigenesis [44]. In this context, CRISPR/Cas9-mediated *NF2* gene editing efficiently disrupted protein expression in PTC cells but without altering cell growth [45]. However, enforced expression of NF2 in ATC cells inhibited cell proliferation, migration and invasion. Interestingly, *NF2* mutagenesis (nonsense mutation S288* and Q470*) restored cell growth in ATC cells, indicating a role for the *NF2* mutations in BRAF^V600E^ -mutated ATCs [45]. 

Similarly, NF1 is a negative regulator of receptors such as RAS and participates in resistance in aggressive tumors. The murine Hras-driven transgenic model (*Tpo-Cre/Hras^G12V^/p53^flox/flox^*) develops poorly differentiated thyroid cancer and ATC that are temporarily responsive to farnesyltransferase inhibitor Tipifarnib [46]. Acquired resistance to this inhibitor is associated with the activation of Ras downstream signaling due to *NF1* truncating mutations. Indeed, the CRISPR/Cas9-mediated biallelic loss-of-function mutation in *Nf1* resulted in an approximate fivefold increase in resistance to Tipifarnib in a murine *Hras;p53* PDTC cell line [46].

Additionally, *EIF1AX*, is another gene that cooperates with MAPK, specifically with RAS mutation in advanced thyroid cancer, and is frequently mutated at a hotspot splice-acceptor site upstream of exon 6 (A113 splice) [47]. An *EIF1AX-*A113-splice mutation with CRISPR/Cas9 knock-in and a donor template via HDR (reviewed in Section 2.3 Mechanism behind Gene Editing) induced cell transformation in normal thyroid cells and increased colony formation in ATC cells [47]. Interestingly, the correction of *EIF1AX-*A113-splice mutation to wild-type sequence using CRISPR/Cas9 knock-in reduced the colony formation effect in ATC cell line C643.

#### 4.1.2. Cancer Cell Migration and Invasion

One hallmark of cancer is the ability of tumor cells to migrate, invade adjacent tissues, and metastasize, which increases the probability of tumor recurrence and cancer-related death. In this process, transcriptional and posttranscriptional modifications occur in the cells that may involve classical EMT but may also involve partial EMT [48]. In thyroid cancer, ATC is the most aggressive, highly metastatic, and lethal form of disease [49]; however, in PTC, lymph node metastases are frequently detected [50].

Platelet-derived growth factor receptor-α (PDGFRA) is a proangiogenic factor that regulates tumor growth and metastasis and is highly expressed in thyroid cancer, correlating with poor survival [51]. Gene editing of *PDGFRA* with CRISPR/Cas9 in aggressive thyroid cancer cells blunted PDGFRA expression, reduced cell invasion and reversed EMT genes expression (reduced *VIM*, *SLUG*, and *N-Cadherin* and induced *E-cadherin*). Moreover, *PDGFRA* gene editing reduced spontaneous metastasis to the lung when injected in immunocompromised mice, indicating a beneficial role for PDGFRA inhibitors, such as imatinib to treat aggressive thyroid cancer [51].

The hematological and neurological expressed 1 (*HN1*) gene is involved in microtubule dynamics and is highly expressed in aggressive thyroid cancer that is associated with poor prognosis characteristics such as age, nodal metastasis and tumor stage [52]. Additionally, *HN1* gene disruption with CRISPR/Cas9 markedly reduced cell migration and invasiveness in ATC cell lines in vitro and blunted tumor growth in vivo. Moreover, CRISPR/Cas9 mediated knockdown of *HN1* was accompanied by EMT gene expression reduction and reactivation of epithelial genes. This effect was associated with the loss of HN1 interaction with Stathmin (STMN1), which blocks a-tubulin acetylation [52]. 

Mitofusin 2 (MFN2), a mitochondrial dynamin-like GTPase that regulates mitochondrial stability and cell metabolism, is expressed at low levels in aggressive thyroid cancer and correlates with BRAF^V600E^-like/low thyroid differentiation tumors [53]. Targeting *MFN2* gene with CRISPR/Cas9 resulted in loss of MFN2 expression in the RAS-mutated ATC cell line Cal62 and induced cell invasion in vitro. Moreover, *MFN2* knockout induced EMT by activating ZEB1, SLUG and N-cadherin gene expression. On the other hand, overexpression of MFN2 reduced EMT genes in ATC cells, reduced cell invasion and migration in vitro, and reduced tumor formation in vivo [53].

Another interesting gene is *BAG5* (BAG cochaperone 5), which is upregulated in PTC and whose expression is associated with fibronectin 1 (FN1) [54]. The depletion of BAG5 protein with CRISPR/Cas9-gene editing reduced cell migration and invasion in thyroid-cancer cells. This effect is associated with FN1 protein downregulation that is negatively targeted by upregulation of miRNA *miR-144-3p*, which is repressed indirectly by BAG5 [54]. 

Most of the studies cited in this thyroid cancer CRISPR section used the plasmid system to express Cas9 nuclease and the sgRNA sequence (as shown in the Section 3 Dry Lab). However, this method generates a stable overexpression of Cas9 and sgRNA that may not be desired in terms of off-targeting potential. Thus, the strategy of transfecting Cas9 RNPs (Cas9 protein complexed with sgRNA explored in Section 3 Dry-Lab: How do I Use CRISPR In Vitro?) results in a temporary presence of Cas9 complex (24–48 h) to edit the desired target, thus minimizing off-targets. This approach was used to investigate the role of LIMD2 in thyroid cancer metastasis. High levels of LIMD2 are detected in metastatic PTC and CRISPR/Cas9 gene editing of *LIMD2* in thyroid cancer cell lines BCPAP and TPC1 reduced cell invasion and modified cell polarity [55]. Moreover, LIMD2 knockout led to the dysregulation of phosphoprotein cascade, which included inducers of the EMT process, such as SLUG and TWIST.

#### 4.1.3. Cell Metabolism, Drug Resistance and Apoptosis

The investigation of thyroid-cancer-cell metabolism by unraveling its metabolic components may contribute to the identification of new therapeutical targets. Moreover, CRISPR/Cas9 can provide a potent tool to target specific genes involved in drug specificity or resistance, and improve cancer treatment.

Drug screening focusing on transcriptional dysregulation revealed ATC cells’ responsiveness to THZ1 inhibitor, leading to strong reduction in cell viability and apoptosis [56]. The main target of THZ1 is CDK7, a cell cycle progression that phosphorylates several substrates including RNA polymerase II activation sites. High levels of CDK7 correlated with poorer prognosis and Ki67 levels in ATC, and targeting *CDK7* with CRISPR/Cas9 resulted in reduction of cell viability, colony formation, G2/M cell cycle arrest and cell apoptosis [56]. Similarly, ATC responds to the treatment with THZ531, an inhibitor that blocks the activity of CDK12 (cyclin dependent kinase 12) in controlling gene transcription [57]. Furthermore, THZ531 reduced CDK12-mediated phosphorylation of RNA pol II and transcriptional elongation, and induced cell growth arrest, apoptosis, and reduction in colony formation in ATC cells. This effect was mimicked by the disruption of the *CDK12* gene using CRISPR/Cas9 [57]. 

In the same context of kinase inhibitors, the treatment of ATC cells with lestaurtinib, an inhibitor of JAK2, resulted in cell cycle arrest and reduction of cell migration [58]. Indeed, CRISPR/Cas9-mediated gene editing of *JAK2* in anaplastic thyroid cancer cells reduced cell proliferation but treatment with lestaurtinib resulted in incremental inhibition, indicating additional target genes or pathways [58].

One interesting gene involved in cell metabolism is the intraflagellar transport 88 (IFT88) gene that participates in primary cilia assembly, a sensory organelle that sends signals from the cell membrane to the control cell metabolism. Loss of *IFT88* gene function using CRISPR/Cas9 resulted in metabolism dysregulation by altering mitochondria function (oxidative phosphorylation) and dynamics (fragmentation) in thyroid cancer cells. Moreover, *IFT88* gene editing reduced cell proliferation, migration, and invasion in ATC cell line [59] 

In hypoxia conditions, NAD(P)H oxidase 4 (NOX4), a mitochondria NOX, is responsible for generating mitochondrial ROS to stabilize HIF1alfa and increase glycolysis and cell growth [60]. The activation of NOX4 is dependent on p22phox, and the depletion of p22phox with CRISPR/Cas9 gene editing leads to a reduction in NOX4 levels. Consequently, mitochondrial ROS generation reduced and destabilized HIF1a-induced glycolysis in hypoxia condition necessary for PTC cell line proliferation [60].

#### 4.1.4. Epigenetics

Chromatin accessibility changes due to posttranscriptional modification in histones and DNA per se may lead to changes in gene expression. At the histone levels, the effect of methylation and acetylation depends on the histone tail position with transcription activation output for acetylation at H3K4 or methylation at H3K36, and transcriptional inhibition for trimethylation at H3K27, for example [61].

High levels of histone deacetylases (HDAC) were detected in PTC and ATC, and the blockage of HDAC function with specific inhibitors induced apoptosis in the transformed thyroid cells [62] and redifferentiation by restoring genes, such as *NIS* (*SLC5A5*), in ATC cells [63]. Moreover, the double-knock out of *HDAC1* and *HDAC2* using CRISPR/Cas9 showed a strong induction of histone acetylation, cell cycle arrest, and apoptosis in ATC cells [64]. Another protein involved in histone acetylation and chromatin accessibility is Bromodomain containing 4 (BRD4) [65]. The CRISPR/Cas9-mediated *BRD4* gene editing resulted in reduced cell viability and proliferation by downregulating c-Myc and cyclin D1 in PTC cells [66]. Moreover, this effect was mimicked by ARV-825, a specific inhibitor of BRD4, in vitro and resulted in tumor growth inhibition in vivo.

Histone methylation is another mechanism involved in gene regulation. For example, ASH1, such as histone lysine methyltransferase (ASH1L), a member of Trithorax group proteins that usually activates gene expression, dimethylates histone H3 at lysine 36, forming H3K36me2. Furthermore, ASH1L is highly expressed in ATC and is regulated by *miR-200b-3p*, a miRNA poorly expressed in this type of cancer. *ASH1L* knockout with CRISPR/Cas9 caused reduction of ATC cell growth in vitro and significantly reduced tumor growth in vivo, but it caused extensive loss of H3K36 dimethylation, especially at *CCAT1* lncRNA [67]. Indeed, *CCAT1* lncRNA knockdown mimicked the *ASH1L* knockout effects in cells, indicating a role of this lncRNA in ATC biology.

Importantly, the CRISPR/dCas9 system can modulate epigenetics (explored in Section 2.4 New Applications for CRISPR/Cas: Tailoring Cas Activity) when dCas9 is fused with chromatin activators (e.g., VPR) or inhibitors (e.g., KRAB) that modify chromatin accessibility in the sgRNA-targeted region, and result in the induction or repression of gene expression, respectively.

### 4.2. Targeting Noncoding Genes in Thyroid Research

In the context of gene-editing noncoding RNAs, the literature on thyroid research is still scarce, with few papers about small RNAs/microRNAs, and long noncoding RNAs. The strategy of gene editing is particularly interesting to blunt the expression of oncogenic miRNAs in thyroid cancer. MiRNAs are small non-coding RNAs (18–22 nt) that regulate target mRNA expression post-transcriptionally by interacting with the 3′-unstranslated region (UTR); furthermore, miRNAs may exert oncogenic or tumor suppressor roles according to the target mRNAs [34]. Among the deregulated miRNAs, *miR-146b-5p* is the most overexpressed in papillary thyroid cancer and is associated with aggressive clinical-pathological characteristics such as extrathyroidal extension [68]. Moreover, a cluster of miRNAs, *miR-17-92* that transcribes seven mature miRNAs, is also linked to aggressive tumors [69] and is overexpressed in anaplastic thyroid cancer [69,70].

Our group used the CRISPR/Cas9n system with mutant nickase Cas9n, which requires a double-sgRNA strategy to target DNA, to disturb the structure of a miRNA gene in two different studies, one targeting *miR-17-92* and the other targeting *miR-146b-5p* [71,72]. First, to disrupt the expression of the *miR-17-92* cluster, we edited a particular region in *MIR17HG* (the host gene for *miR-17-92*) by targeting a double-sgRNA+Cas9n to the proximal region of *miR-17* that contains a splicing site [73]. As a result, we observed a strong reduction in the expression of all cluster miRNAs in ATC cell line KTC2, especially *miR-17* which was closer to the gene-editing site [71]. As a functional effect, ATC cells showed reduction in cell viability, cell migration and colony-formation ability, but restored the expression of thyroid transcription factors, PAX8 and NKX2-1, and responsiveness to TGFβ antimitogenic cytokine [71]. 

In a further study, we showed that CRISPR/Cas9n can be directed to target and edit the *MIR146B* gene, which transcribes the most overexpressed miRNA in thyroid cancer, *miR-146b-5p*. We also used the double-sgRNA strategy to target the *miR-146b-5p* sequence and disrupt the precursor hairpin structure. We observed strong reduction in *miR-146b-5p* mature level that was sufficient to result in important functional effects in the ATC cell line KTC2, such as impairment of cell proliferation, cell migration and cell growth in immunocompromised mice [72]. These results indicate the importance of miRNAs for thyroid cancer biology, and that targeting miRNAs with CRISPR/Cas9n may become a promising approach for aggressive thyroid cancer.

In the context of small noncoding RNAs (<200 nt), *nc886* (officially called vault RNA 2-1) is a small 101-nt RNA transcribed by RNA polymerase III with intriguing similarities to vault RNA and pre-miRNA that are still under investigation [74]. Nevertheless, *nc886* acts as a direct inhibitor of protein kinase R (EIF2AK2: eukaryotic translation initiation factor 2-alpha kinase 2), thus regulating cell growth [75]. High levels of *nc886* are associated with lymph-node metastasis and tumor aggressiveness in thyroid cancer [76]. The temporary knock-down of *nc886* with siRNA results in cell death and activation of PKR (phosphor-PKR) in thyroid cells. To study the effect of permanent *nc886* deletion, a segment of approximately 280 bp that contained the *nc886* transcript was removed from the genome by using a double-sgRNA strategy with wild-type Cas9. Because of *nc886* knockout, thyroid cells exhibited an impairment of the tumoral phenotype such as a reduction in cell proliferation, migration, and invasion associated with blockage of transcriptional programs (target gene expression) that support an oncogenic role [76]. 

Long-noncoding RNAs (>200 nt) are also an important class of noncoding RNAs that exert functional roles in thyroid cancer, with a diverse range of transcriptional and posttranscriptional effects [77]. The application of CRISPR/Cas9-directed gene editing is also possible in long-noncoding transcripts, for example, in the 2346-bp long noncoding RNA of small nucleolar host gene 3 (*SNHG3*). The analysis of the PTC gene sets revealed low expression of *SNHG3* compared to normal thyroid which was associated with poor clinical outcomes, especially recurrence-free survival [78]. To investigate the effect of *SNHG3*, CRISPR/Cas9 mediated gene disruption was employed in thyroid cancer cell lines (SNHG3-KO) and resulted in strong reduction of *SNHG3* levels and induced pro-tumorigenic effects, such cell proliferation, migration and invasion [78]. Moreover, there was an important acceleration of tumor formation of *SNHG3*-KO cells in immunocompromised mice, and this effect was associated with AKT/mTOR/ERK pathway activation.

## 5. Perspectives

The consolidation of the CRISPR/Cas9 methodology, a versatile tool for gene editing and gene modulation (Cas9 mutants), has paved the way for the rapid expansion in the understanding of the molecular mechanism of cell signaling in thyroid cancer as summarized in Table 3. Most of the studies presented in this review utilize CRISPR/Cas9 methodology to blunt the expression of a target gene. However, further applications, such as gene correction using CRISPR/Cas9 +donor DNA (HDR) and gene-expression modulation (dCas9-KRAB or dCas9-VPR, for example), compose an array of applications that have the potential to be implemented in future cancer treatments. 

One concern in the technique is the correct delivery of CRISPR/Cas to cells (in the context of an organism), either by viral particles, plasmids or nanoparticles [79], remaining a technical difficulty that regards most of the genetic therapy. As an encouraging application of CRISPR/Cas in cancer, we can mention immunotherapy with the first trials of CRISPR-edited T cells in lung cancer, and in in advanced refractory cancer [80,81]. Somewhat similar, both trials rely on the CRISPR/Cas9 gene editing of the human *PD1* gene or a multiplex strategy to edit *TRAC*, *TRBC*, and *PDCD1* genes in autologous T-cells extracted from cancer patients in order to enhance the antitumor effect. The infusion of these CRISPR-modified T-cells into patients proved to be safe in both studies, and already showed antitumor effect in the phase I study [80]. 

Nevertheless, the rapid expansion of CRISPR/Cas applications in vitro and in vivo may provide the correct timing for a global effort to further develop the delivery strategies in terms of specificity and safety, while mitigating the off-targeting effects. This would encourage a safer next step for the treatment of aggressive cancers, including thyroid cancer.

## Abbreviation

Glossary of CRISPR/Cas system nomenclature.
**Abbreviation/Term****Meaning****Function/Use**CRISPRCluster of Regularly Interspaced Palindromic RegionsRegion of repetitive sequences in the bacteria genome that contains genomic sequence to target foreign DNACas genesCRISPR-associated genesGene associated with the CRISPR locuscrRNACRISPR RNASmall RNA transcribed from CRISPR locustracrRNATransauxiliary CRISPR RNASmall RNA that interacts with crRNA for proper processing and foldingPAMProtospacer adjacent motifDNA sequence present in the non-complementary DNA strand that is essential to Cas9-directed DNA cleavagesgRNASingle-guide RNAArtificial fusion of crRNA + tracrRNAExpression PhaseExpression PhasecrRNA is expressed from CRISPR locus and interacts with tracrRNAAdaptation PhaseAdaptation PhaseForeign DNA sequence is incorporated into CRISPR locus by Cas proteinInterference PhaseInterference PhaseRecognition of invader DNA by crRNA + tracrRNA complexed in Cas9 and cleavage of target DNANUC lobeNuclease lobeCas9 lobe that cleaves double-strand DNA using HNH and RuvC nuclease domainsREC lobeRecognition lobe Cas9 lobe that contains three alpha-helical domainsNHEJNon-homologous end joiningA error-prone mechanism of DNA repair that is very efficient is rejoining double-strand breaks HDRHomology-directed recombinationA proof-read mechanism of DNA repair that relies on a DNA donor templateCas9Cas9 nucleaseClass II endonuclease that acts in the recognition and interference phase.Cas9nCas9 nickaseA mutant Cas9 that cleaves only one strand of DNA due to mutations in either RuvC or HNH domain.dCas9Dead Cas9A mutant Cas9 that does not cleave DNA (dead) due to mutations in both RuvC and HNH domainsCRISPRiCRISPR interferenceMethodology that uses dCas9 fused to transcriptional inhibitor to repress target gene expressionCRISPRaCRISPR activationMethodology that uses dCas9 fused to transcriptional activators to induce target gene expressionKRABKrüppel-associated box KRAB domain of Kox1 gene fused to dCas9 in order to inhibit the expression of target geneVPRVP64-p65-RtaIn tandem fusion of VP64-p65-Rta activator domains in dCas9 to induce target gene expression

## Figures and Tables

**Table 1:** Class2 Cas nucleases comparison.

Organism	CRISPR Nuclease	CRISPR Type	PAM Sequence (5’-3’)	Size (Aminoacids)	Aim	Reference
*Streptococcus pyogenes*	SpCas9	Class2, Type II	NGG	1368 aa	Gene editing	[2]
*Staphylococcus aureus*	SaCas9	Class2, Type II	NNGRR or NGRRN	1053 aa	Gene editing	[9]
*Neisseria meningitidis*	NmeCas9	Class2, Type II	NNNNGATT	1082 aa	Gene editing	[10]
*Streptococcus thermophilus*	StCas9	Class2, Type II	NNAGAAW	1409 aa	Gene editing	[11]
*Lachnospiraceae bacterium*	LbCas12a (former Cpf1)	Class2, Type V	TTTV	1228 aa	Gene editing	[12]
*Deltaproteobacteria*	DpbCasX (Cas12e)	Class2, Type II	TTCN	996 aa	Gene editing	[13]

**Table 2:** Cas9 and the mutated variants used for gene editing and gene modulation.

Cas Protein	RuvC Domain	HNH Domain	Aplications	References
Cas9	Wild-type	Wild-type	Gene editing	[2,18]
Cas9n	D10A	Wild-type	Gene editing	[17,19]
Cas9n	Wild-type	H840A	Gene editing	[17]
dCas9	D10A	H840A	Gene modulation, DNA imaging, etc.	[20,21,22]

**Table 3:** Applications of CRISPR/Cas9 gene editing in thyroid cancer.

Section	Targeted Gene	Cell Line/Animal Model	Thyroid Cancer Histotype	Type of Cas	Main Observations	Reference
*MAPK pathway*	*Twist3*	Zebrafish Tg-BRAF-TOM	PTC	Cas9	Partial restoration of thyroid follicular structure in zebrafish.	[41]
	*EGFR*	SW579	ATC	Cas9	Cell cycle arrest and reduction of colony formation in ATC cell line.	[42]
	*NF2*	KTC1	PTC	Cas9	Depletion of NF2 did not change PTC cell growth; Overexpression reduced cell growth.	[45]
	*Nf1*	Murine Hras^G12V^/p53^flox/flox^ cell line	PDTC	Cas9	Induction of resistence to Tipifarnib.	[46]
	*EIF1AX*	CAL62, TTA1 and C643	ATC	Cas9	EIF1AX A113-splice mutation increased colony formation; while A113-splice correction reduced colony formation.	[47]
*Cancer cell invasion and migration*	*PDGFRA*	SW579	ATC	Cas9	Restored epithelial gene expression and reduced cell invasion in vitro; impaired lung metastasis in vivo.	[51]
	*HN1*	8505C and CAL62	ATC	Cas9	Reduced cell invasion and migration in vitro; Inhibited tumor growth in vivo.	[52]
	*MFN2*	Cal62	ATC	Cas9	Promotes epithelial-mesenchymal transition and invasion of ATC cells.	[53]
	*BAG5*	IHH4	PTC	Cas9	Reduced cell invasion and migration by downregulation of FN1 via miR-144-3p.	[54]
	*LIMD2*	BCPAP/TPC1	PTC	Cas9	Reduced cell invasion and EMT markers while improved cell polarity.	[55]
*Cell metabolism, drug resistence and apoptosis*	*CDK7*	Cal62	ATC	Cas9	Reduced colony formation and cell viability, while induced cell cycle arrest and apoptosis.	[56]
	*CDK12*	Cal62	ATC	Cas9	Reduced colony formation and cell growth, while induced apotosis.	[57]
	*JAK2*	KMH2/CAL62	ATC	Cas9	Reduced cell proliferation.	[58]
	*IFT88*	8505C	ATC	Cas9	Reduced cell proliferation, invasion and migration, dysregulated cell metabolism.	[59]
	*p22phox/CYBA*	TPC1	PTC	Cas9	Reduced mitochondrial ROS generation and impaired PTC cells proliferation in hypoxia.	[60]
*Epigenetics*	*HDAC1/ HDAC2*	SW579	ATC	Cas9	Enhanced histone acetilation levels and induced cell cycle arrest and apoptosis.	[64]
	*BRD4*	TPC1	PTC	Cas9	Reduced cell viability and cell proliferation.	[66]
	*ASH1L*	BHT-101	ATC	Cas9	Reduced cell growth in vitro and tumor growth in vivo.	[67]
*Targeting noncoding genes*	*MIR17HG*	KTC2	ATC	Cas9n	Reduced cell viability, migration and colony formation, and improved cell differentiation.	[71]
	*MIR146B*	KTC2	ATC	Cas9n	Reduced cell proliferation, viability and migration in vitro; Reduced tumor growth in vivo.	[72]
	*nc88/VTRNA2-1*	C643	ATC	Cas9	Reduced cell proliferation, migration and invasion.	[76]
	*SNHG3*	BCPAP/TPC1	PTC	Cas9	Induced cell proliferation, migration and invasion in vitro; Induced tumor growth in vivo.	[78]

Abbreviations—PTC: papillary thyroid cancer; PDTC: poorly differentiated thyroid cancer; ATC: anaplastic thyroid cancer.
